# An Accurate CFD-FEM Model for the Thermal Stress of the Simulation of Selective Laser Melting

**DOI:** 10.3390/ma19010022

**Published:** 2025-12-20

**Authors:** Yilai Chen, Xuezhi Zhang, Anguo Huang, Shengyong Pang, Lvjie Liang

**Affiliations:** 1 School of Mechanical and Electrical Engineering, Wuhan Institute of Technology, Wuhan 430205, China; 2 State Key Laboratory of Materials Processing and Die & Mould Technology, School of Materials Science and Engineering, Huazhong University of Science and Technology, Wuhan 430074, China

**Keywords:** CFD-FEM method, selective laser melting, stress field, numerical simulation, additive manufacturing

## Abstract

Selective laser melting (SLM) is a 3D printing technology for precision manufacturing. Owing to its high forming accuracy, parts fabricated by SLM can often be used directly without secondary machining. Consequently, the stress field in the structure, especially local stress concentration in small regions, is of great importance. Building on our previous work, this study proposes an accurate and efficient thermo-mechanical analysis method that combines a computational fluid dynamics (CFD) model and a finite element method (FEM) model for stress prediction in micrometer-scale SLM. Compared with the conventional element birth–death method, the present model more faithfully reproduces the SLM process and the post-solidification morphology and stress distribution. Numerical simulation of a single-track TC4 scan shows that pronounced surface undulations and lack-of-fusion regions exhibit significant stress concentration: the local residual stress can reach approximately 900 MPa, whereas regions with relatively smooth surface geometry exhibit stresses of about 650 MPa. This indicates a clear positive correlation between surface quality and stress concentration. The results provide a new theoretical basis for understanding defect formation mechanisms, spatial stress distribution, and scan-path optimization in SLM components.

## 1. Introduction

Selective laser melting (SLM) is an additive manufacturing (AM) technology that uses a high-energy laser beam to selectively melt metal powder layer by layer for rapid fabrication of components with complex geometries [1,2,3,4]. Owing to its advantages of high relative density, simple process, wide range of processable materials, and good forming quality, SLM has been widely used in aerospace, automotive, and medical applications [5,6]. However, during fabrication, the material experiences cyclic and severe thermal loading with steep thermal gradients, which easily leads to severe local residual stresses, distortion, and even cracking [7,8]. Recent review papers have pointed out that SLM involves multiple physical processes, including laser–material interaction, melt-pool flow, metal evaporation, and solidification. These processes evolve in a strongly coupled manner on very short time scales. Relying solely on experiments is not only costly but also makes it difficult to fully observe mechanisms such as stress evolution and defect formation; therefore, numerical simulation has become an indispensable tool for studying SLM processes [9,10,11]. These studies indicate that only by accurately accounting for melt-pool flow, surface morphology, and temperature evolution in simulations can residual stress and defect distributions be reliably predicted.

Over the past decades, extensive research has been conducted on the numerical simulation of SLM. In terms of stress prediction, numerous studies have developed residual-stress models at different scales based on FEM. Hussein et al. [12] used the ANSYS Parametric Design Language (APDL) to build a single-layer model and simulated the temperature and stress fields of 316L stainless steel during SLM. Nagesha et al. [6] developed a thermo-mechanical FEM model and compared the simulated residual stresses with experimental measurements; the numerical results were in good agreement with the experiments and followed the same trend. Kayacan et al. [13] showed that residual stresses mainly occur at the top surface, the substrate interface, and the bonding surface of the part. Gu et al. [14] developed a three-dimensional transient FEM model to predict the stress distribution in SLM-fabricated components and found that the maximum residual stress appears at the ends of the first and last scan tracks. Although these FEM models can effectively describe the overall stress distribution, most studies use equivalent heat sources or simplified melt-pool shapes and do not fully consider melt-pool flow and powder melting. As a result, their predictive capability at the micrometer scale and for local stress concentration is limited.

In the field of fluid-dynamics simulation of SLM, CFD models have been widely used to investigate melt-pool morphology and temperature distribution. Liu et al. [15] proposed a rapid analytical model for SLM melt-pool geometry that considers both the thermal balance at the leading edge of the pool and the characteristic pool shape resulting from the interaction between recoil pressure and liquid-metal flow. Ren et al. [16] established a high-fidelity CFD model and introduced a temperature-dependent absorption law to overcome the bias caused by the widely used Fresnel absorption assumption. Gu et al. [17] developed a comprehensive multi-track modeling framework to simulate the evolution and morphology of melt pools formed by various materials deposited in the same and different layers in multi-layer, multi-material SLM, and investigated phenomena such as balling, keyhole depression, and lack of inter-layer fusion. Wang et al. [18] used a multiphysics thermal–fluid model to reveal the formation mechanism of keyhole pores, including the generation of transient bubbles, their entrapment at the solidification front, and their motion in the melt pool, and reported that pore sizes can reach tens of micrometers under specific process parameters.

Although CFD models can capture melt-pool morphology and flow, and FEM models can, to some extent, simulate stress distribution, most existing studies have not effectively coupled the two. Common stress-prediction models often neglect the influence of powder melting and melt-pool flow on stress evolution.

To address this issue, this work extends our previous CFD-FEM coupling approach for wire-feeding-based laser additive manufacturing (WFLAM) [19] to the SLM process and proposes a high-fidelity CFD-FEM coupled model for SLM. Unlike the previous model, which mainly focused on millimeter-scale wire-deposition processes without involving powder behavior or micrometer-scale melt pools, the present model incorporates powder melting, melt-pool flow, and Marangoni effects in the CFD module. By using an improved numerical mapping algorithm, melt-pool surface morphology and temperature-field information are transferred with high accuracy to the FEM mesh, enabling prediction of local residual stress distribution.

## 2. The Simulation Method

### 2.1. CFD-FEM Multiphysics Coupling Framework

**Figure 1:**
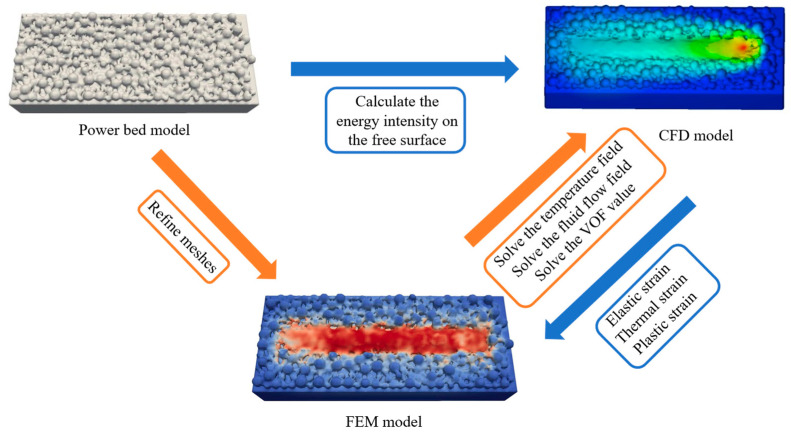
Overall CFD-FEM modeling framework.

A CFD-FEM multiphysics coupling framework is employed to simulate melt-pool evolution and residual stress during SLM, as shown in Figure 1. First, a CFD model is used to solve the melt-pool flow, free-surface evolution, and transient temperature field induced by laser–powder interaction. The temperature field and free-surface morphology obtained from CFD are then mapped onto the FEM mesh for stress analysis. Compared with conventional FEM models using equivalent heat sources, the present approach retains the dynamic melt-pool morphology and temperature evolution with high fidelity, thereby improving the accuracy of residual-stress prediction. Based on our previous work [19], the CFD-FEM coupling framework is extended from wire-feeding deposition to powder-bed SLM, and a more refined mapping strategy is adopted to handle the micrometer-scale dynamic free surface in SLM.

### 2.2. The CFD Model

Following previous studies, the CFD model is formulated as a three-dimensional incompressible flow model that considers mesoscale heat conduction and convective heat transfer [20,21,22,23]. A CFD solver is used to compute the melt-pool flow, phase change, heat and mass transfer, and melt-track evolution induced by laser–powder-bed interaction [24,25]. The governing equations of mass, momentum, and energy in the reference frame can be written as follows [26,27,28,29]:

(1) $$\frac{\partial \bar{\rho }}{\partial t}+\nabla \cdot \left(\bar{\rho }u\right)=0$$

(2) $$\frac{\partial \bar{\rho }u}{\partial t}+\nabla \cdot \left(\bar{\rho }u⊗u\right)=-\nabla p+\nabla \cdot \left(\mu \nabla u\right)-K_{c}\left[\frac{{\left(1-f_{l}\right)}^{2}}{f_{l}^{3}+c_{K}}\right]u+\bar{\rho }g+F_{st}+F_{M}+P_{r}$$

(3) $$\frac{\partial \bar{\rho }h}{\partial t}+\nabla \cdot \left(\bar{\rho }uh\right)=\nabla \cdot \left(D_{f}\nabla h\right)+q_{abs}-q_{loss}\left|\nabla \alpha_{1}\right|\frac{2\bar{\rho }}{\rho_{s}\left(1-\gamma \right)+\rho_{l}\gamma +\rho_{g}}$$

where $\bar{\rho }$, t, u, p, μ, $-K_{c}\left[\frac{{\left(1-f_{l}\right)}^{2}}{f_{l}^{3}+c_{K}}\right]u$, $K_{c}$, $f_{l}$, $c_{K}$, g, $F_{st}$, $F_{M}$, $P_{r}$, h, $D_{f}$, $q_{abs}$, $q_{loss}$, $\nabla \alpha_{1}$, $\rho_{s}$, $\rho_{l}$, $\rho_{g}$, γ represent the average fluid density, time, fluid velocity vector, fluid pressure, dynamic viscosity, damping term in the liquid-phase mixture zone, permeability coefficient, volume fraction, a small constant, gravitational acceleration, surface tension, Marangoni force, recoil pressure, specific enthalpy of the fluid, thermal conductivity, absorbed heat, lost heat, phase field variable, solid density, liquid density, gas density, and phase fraction parameter, respectively.

The Marangoni force is computed from the temperature-dependent surface-tension gradient and can significantly affect thermal convection in the melt pool [30]. Under high-energy-density SLM conditions, the laser induces rapid local metal evaporation; the recoil pressure generated by local evaporation acts on the free surface and is one of the main driving forces for melt-pool depression [31]. Among them, $F_{st}$, $F_{M}$, and $P_{r}$ can be expressed as follows [32,33,34,35]:

(4) $$F_{st}=\sigma \kappa n\frac{2\bar{\rho }}{\rho_{s}\left(1-\gamma \right)+\rho_{l}\gamma +\rho_{g}}$$

(5) $$F_{M}=\frac{d\sigma }{dT}\left[\nabla T-n\left(n\cdot \nabla T\right)\right]\left|\nabla \alpha_{1}\right|\frac{2\bar{\rho }}{\rho_{s}\left(1-\gamma \right)+\rho_{l}\gamma +\rho_{g}}$$

(6) $$P_{r}=0.54P_{0}\exp\left[\frac{L_{v}M\left(T-T_{v}\right)}{RTT_{v}}\right]n\left|\nabla \alpha_{1}\right|\frac{2\bar{\rho }}{\rho_{s}\left(1-\gamma \right)+\rho_{l}\gamma +\rho_{g}}$$

where σ is the surface tension coefficient, κ is the interface curvature, n is the interface normal vector, $\frac{d\sigma }{dT}$ is the temperature coefficient of surface tension for TC4 material, $P_{0}$ is the ambient pressure, $L_{v}$ is the latent heat of evaporation, $T_{v}$ is the evaporation temperature, M is the molar mass, and R is the universal gas constant.

A volume of fluid (VOF) method is employed to track the interface between metal and gas. In the discrete sense, the interface thickness is typically confined to 1–2 grid cells, and geometric interface reconstruction is adopted to reduce numerical diffusion, allowing the steep geometry of keyhole walls and free surfaces to be represented with high clarity. The liquid fraction varies linearly with temperature as follows [36]:

(7) $$f_{l}=\left\{\begin{array}{cc} 1 & T>T_{l}\\ \frac{T-T_{s}}{T_{l}-T_{s}} & T_{l}\geq T\geq T_{s}\\ 0 & T<T_{s} \end{array}\right.$$

where $T_{s}$ represents the solid-phase temperature of the material, and $T_{l}$ represents the liquid-phase temperature of the material.

Because the liquid fraction is obtained solely by linear interpolation between $T_{s}$ and $T_{l}$ for a given material, the structure of the governing equations and the model formulation do not depend on a specific material. Therefore, the model can be readily extended to steels, titanium alloys, and Ni-based alloys by simply replacing the material parameters.

The governing equations for the volume of fluid method are as follows [37]:

(8) $$\frac{\partial F}{\partial t}+\vec{U}\cdot \nabla F=0$$

where F is defined as the volume of the fluid.

### 2.3. The Dynamic Interface Element Method

In SLM, powder is melted to form a melt pool, which eventually solidifies into the printed track. During this process, the metal state continuously changes. The complex evolution of the free surface has a pronounced influence on stress, for example, by causing stress concentration near pores.

**Figure 2:**
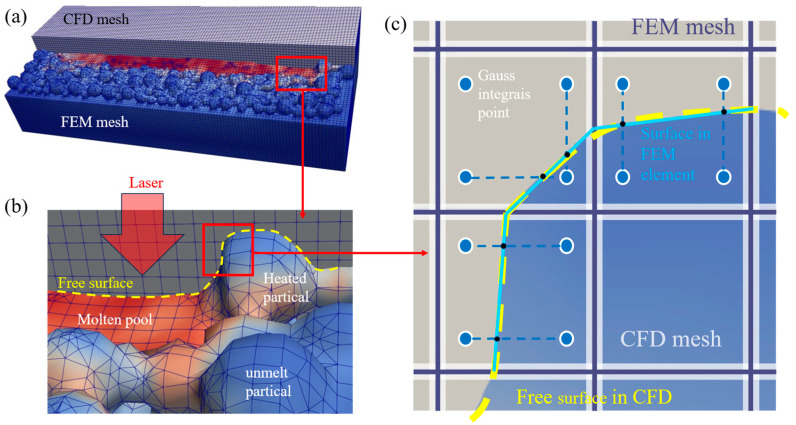
Interpolation algorithm of the dynamic interface: (**a**) CFD mesh and FEM meshes; (**b**) melt-pool region; (**c**) interpolation within an FEM element.

To accurately describe the influence of the free surface on stress, a coupled model is proposed to study stresses in SLM. The CFD model is used to simulate the thermal field and dynamic free surface, and the FEM model is used to compute the thermo-mechanical field with a dynamic free surface. A key step in the algorithm is the mapping procedure near the free surface. Here, we introduce a new type of element, termed the interface element, which differs from the conventional birth–death element. As illustrated in Figure 2, both the CFD and FEM models employ structured hexahedral meshes of identical size, so that the grids are spatially coincident and the nodes correspond one-to-one. This design greatly improves the accuracy of surface-morphology mapping from CFD to FEM.

In the traditional birth–death element method, the state of each element is determined by its VOF value. By comparing the VOF value with a specified threshold, elements are classified as active or inactive. For instance, elements with a VOF value greater than 0.5 are defined as active, whereas the others are defined as inactive. In the present model, after introducing the interface element, the definitions of active elements, inactive elements, and interface elements are as follows:

(9) $$style=\left\{\begin{array}{c} Active\\ Inactive\\ Interface \end{array}\right.$$

**Figure 3:**
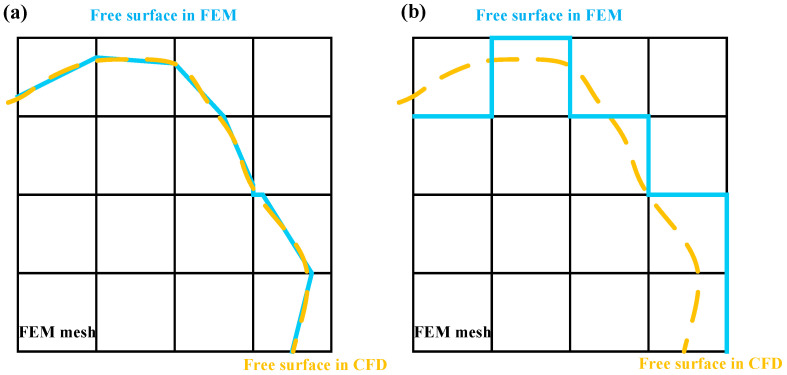
The free surfaces in the CFD and FEM meshes: (**a**) proposed algorithm; (**b**) conventional birth–death method.

With the incorporation of interface elements, the free surface in the FEM model can more precisely approximate the actual physical free surface. As shown in Figure 3, the free surface in the FEM model aligns more closely with that predicted by the CFD model.

### 2.4. The FEM Model Incorporating the Dynamic Interface

Unlike the conventional birth–death method, which simply classifies elements as active or inactive. the present method introduces a transitional element type in the region intersected by the free surface. The VOF value is used to represent the element status. In VOF-based interface capturing, the transition zone where the volume fraction varies between 0 and 1 is usually regarded as the numerical interface region, and the isosurface of f=0.5 is often used to represent the geometric interface position [38]. For example, Lv et al. [39] used the f=0.5 isosurface to represent the free surface in dam-break simulations, and Tsui [40] defined the interface as the f=0.5 isosurface in the CISIT method. Accordingly, the present study also adopts the VOF value f=0.5 for free-surface identification. The VOF value is introduced to represent the birth and death states of the elements. This model considers three distinct cases: (1) f=0.5 represents the interface between the gas and the solid or liquid; (2) f>0.5 represents the solid or liquid phase; and (3) f<0.5 represents the gas phase. The function f presents the volume of fluid.

(10) $$F_{i}=\left\{\begin{array}{cc} 1 & \forall j:f_{i}(j)\geq 0.5\\ 0 & \forall j:f_{i}(j)\leq 0.5\\ \sum \frac{f_{i}(j)}{N} & else \end{array}\right.$$

where *N* denotes the number of nodes in the element, and $F_{i}$ represents the birth and death element coefficient of the FEM element.

During stress analysis, the temperature field is obtained from the CFD solution, so thermal conductivity and specific heat are only used in CFD and no longer participate in the mechanical computation inside FEM. Stress is computed based on the temperature distribution, thermal expansion coefficient, and temperature-dependent elastoplastic constitutive law.

In the production elements, the expression form is consistent with that in the finite element calculation of solid mechanics. The stress–strain relationship is expressed as follows:

(11) $$\left\{d\sigma \right\}=F_{i}\left([D^{p}]\left\{d\varepsilon \right\}-[D^{p}]\left\{\alpha \right\}dT+[D^{p}]\frac{1}{E}\frac{dE}{dT}{\left[D^{e}\right]}^{-1}\left\{d\sigma \right\}dT\right)$$

where $\left[D^{p}\right]$ is the plastic matrix, and $\left[D^{e}\right]$ is the elastic matrix. $\left\{\alpha \right\}$ is the linear expansion coefficient, and E is Young’s modulus.

During stress computation, both the elastic modulus and yield strength are temperature dependent, and a thermo-elastoplastic framework is used. In regions above the liquidus temperature, a very small elastic modulus and yield strength are assigned to approximate liquid behavior. For inactive elements, the thermal conductivity and elastic modulus are set to a minimal value, and the whole structure still participates in the solution of the linear equations as a part of the whole with the minimal form of an elastic body.

For interface elements, a constitutive model in the form of a specific gradient will be derived. In this paper, the hexahedral elements of the first-order scheme are adopted, that is, in each element, the number of Gaussian integration points is 8. The value of VOF at each Gaussian integral point is expressed as

(12) $${f^{\left(FEM\right)}}_{i}=\sum_{m=0}^{8}N_{m}\left(\left\{x_{i,j}\right\}\right)\cdot {f^{\left(CFD\right)}}_{j,m}$$

where ${f^{\left(FEM\right)}}_{i}$ represents the VOF value of the node i in the FEM model, $N_{m}\left(\left\{x_{i,j}\right\}\right)$ denotes the shape function of the hexahedral element in the CFD model, $\left\{x_{i,j}\right\}$ is the local coordinate of the node i under the coordinate system of the element j in the CFD model. ${f^{\left(CFD\right)}}_{j,m}$ indicates the VOF value of the node m of the element j in the CFD model. During the interpolation and mapping of temperature and volume fraction from the CFD mesh to FEM nodes, one-to-one node correspondence and high-density data output from CFD to FEM effectively control local energy distortion.

(13) $${F^{\left(FEM\right)}}_{i}=\sum_{n}\frac{{f^{\left(FEM\right)}}_{i}}{n}$$

where ${F^{\left(FEM\right)}}_{i}$ denotes the VOF value of the element *i* in the FEM model.

In the mapping process, the free surface is transferred directly via the VOF value f=0.5 isosurface without additional smoothing of the morphology. This preserves local curvature features and avoids smoothing sharp depressions caused by interpolation. To maintain temporal continuity of free-surface evolution and temperature distribution during mapping, a small time step is used in the CFD solution so that changes in free-surface position and temperature between consecutive steps are strictly constrained. This significantly mitigates transient jumps in the FEM stiffness matrix and enhances the convergence and stability of the coupled solution.

The stiffness matrix of the element can be expressed as

(14) $${\left[K\right]}^{\left(e\right)}=\int_{v}\left[B^{T}\right]\left[D\right]\left[B\right]dv$$

**Figure 4:**
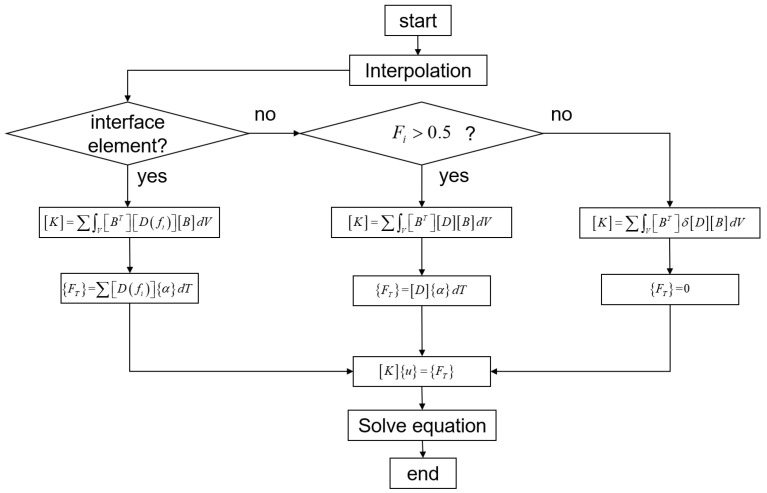
Flowchart of the overall solution process.

The whole structure was solved based on the NR iterative algorithm and the elastic–plastic model, As shown in Figure 4.

## 3. Simulation Case

### 3.1. SLM Process and Operating Parameters

**Figure 5:**
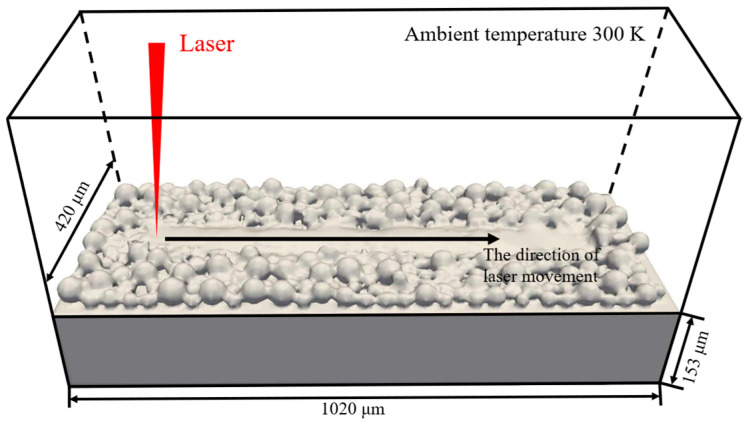
Simulation model.

A single-track TC4 powder-bed melting process is simulated, as shown in Figure 5, to validate the proposed thermal fluid-dynamics model. The simulation domain consists of a substrate of length, width, and height 1020 μm, 420 μm, and 153 μm, respectively, and a powder bed with a maximum powder diameter of 50 μm. This particle size lies within the commonly used range for TC4 metal powders in SLM [41,42]. The domain is sufficiently large to cover the entire melt pool and heat-affected zone, thereby avoiding non-physical reflections near the boundaries and ensuring realistic transient temperature and free-surface evolution.

The laser parameters are listed in Table 1 and fall within the typical process window for TC4 in SLM [43]. The thermal boundary conditions adopt a natural convective heat-transfer coefficient of 20 W/m^2^·K and an emissivity of 0.4, which lie within the experimental parameter ranges commonly used in thermo-mechanical analyses of SLM [44]. Natural convection and radiation are both realistic heat-dissipation mechanisms for metal powder beds. The melt-pool free surface is described using the VOF method. The preheating and ambient temperatures are set to 300 K.

The thermophysical properties of TC4 are summarized in Table 2. To obtain a more accurate stress field, the specific heat, elastic modulus, yield strength, and thermal conductivity of TC4 are treated as temperature-dependent functions rather than constants, allowing a realistic representation of the drastic property variations during rapid heating and solidification.

### 3.2. Simulation Parameters and Meshes

**Figure 6:**
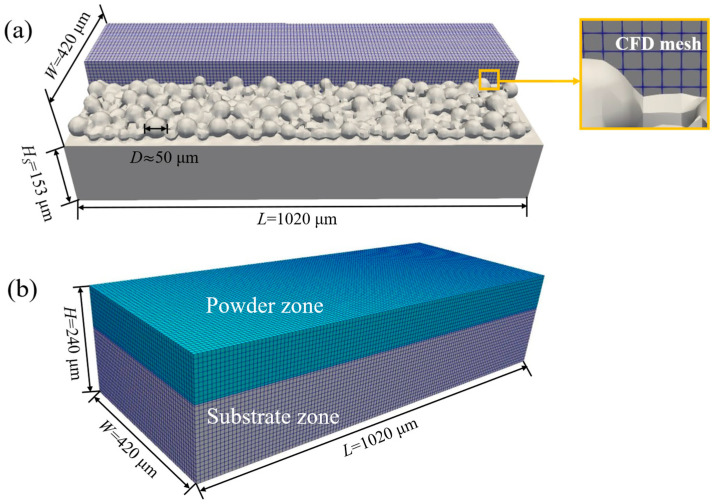
Simulation meshes: (**a**) CFD mesh; (**b**) FEM mesh.

As shown in Figure 6a, the CFD model uses a structured hexahedral mesh with an element size of 8 μm and a total of 228,802 elements. The domain dimensions are 1020 μm × 420 μm × 240 μm. This resolution is sufficient to resolve key SLM melt-pool features, including free-surface variations and temperature gradients. Considering the surface curvature and overall dimensions, using first-order hexahedral elements with an 8 μm edge length offers a good compromise between computational efficiency and accuracy. The default time step is 1 × 10^−8^ s, and the minimum and maximum time steps are 1 × 10^−18^ s and 1 × 10^−6^ s, respectively. The small time step prevents abrupt changes in free-surface position and temperature peaks between consecutive steps, ensuring that the temperature field and morphological evolution transferred to the FEM model vary smoothly over time. Consequently, the stiffness matrix in the FEM model also changes continuously, thereby improving the stability and convergence of the coupled simulation.

The FEM model, shown in Figure 6b, has the same domain size as the CFD model and uses hexahedral elements of the same size, ensuring one-to-one nodal correspondence and high-accuracy data mapping. The laser scan length is 800 μm.

## 4. Results and Discussion

### 4.1. Single-Track Simulation Results

**Figure 7:**
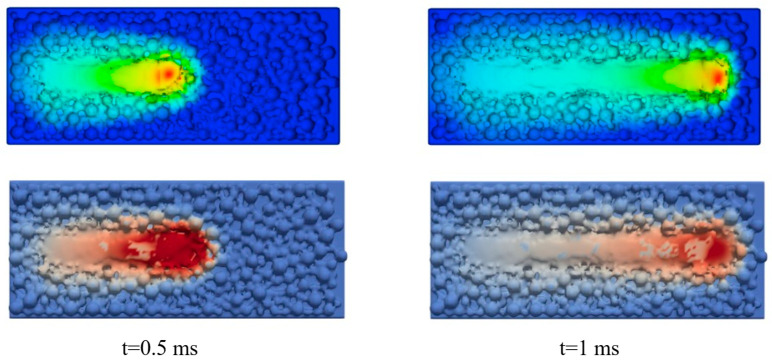
Comparison of temperature distributions between CFD and coupled models at the same time instants: CFD (**top**) and coupled model (**bottom**).

To verify the fidelity of the coupling algorithm in terms of geometry and temperature transfer, the melt-pool free-surface morphology obtained from the CFD model at t = 0.5 ms and t = 1 ms is extracted and compared with the coupled CFD-FEM results, as shown in Figure 7. The free-surface position, surface undulation features, and temperature distribution in the coupled model agree very well with those from the CFD model. This demonstrates that the proposed CFD-FEM mapping scheme can accurately preserve the transient surface morphology and spatial distribution of temperature gradients at each time step.

**Figure 8:**
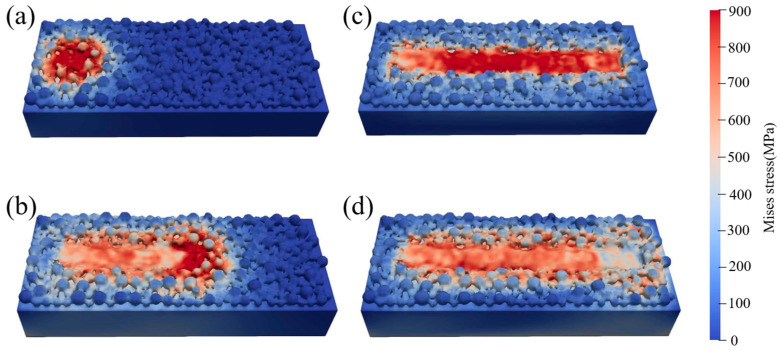
Stress evolution during single-track scanning: (**a**) t = 0 ms; (**b**) t = 0.5 ms; (**c**) t = 1 ms; (**d**) t = 50 ms.

The stress evolution during single-track scanning is shown in Figure 8, where a–c represent the stress evolution during the laser scanning process, and Figure 8d shows the residual stress distribution after the melt pool has cooled to 50 ms. During the laser scanning, as the laser moves, the powder gradually melts to form a scanning track. The stress inside the melt pool is negligible, and only the heat-affected zone around the partially melted powder exhibits a certain stress, approximately 400 MPa. As the heat source moves, the scanning track expands, and the partially melted powder particles bond to the substrate. The most significant shape change occurs at the interface between the powder and the substrate, where stress concentration appears, and the maximum stress can reach 900 MPa. With the gradual decrease in temperature and slow solidification at the welded area, the shrinkage of the melt pool is restricted by the substrate, leading to a certain amount of stress that increases as the temperature decreases. After scanning is completed, the system cools for 50 ms. At this point, tensile stress is at its maximum and most complex, showing continuous “oscillation” phenomena in the morphology, as shown in Figure 8d. Strong stress concentration occurs at the interfaces where the powder bonds to powder, powder bonds to the substrate, and in lower regions of the scanning track, with a maximum value of 900 MPa. In contrast, stress at the relatively high convex positions of the scanning track is only 350 MPa. These extremely high stress peaks may be attributed to the large thermal gradients and cooling rates induced by laser scanning, combined with the high strength and elastic modulus of TC4; similar phenomena have been observed experimentally in some studies [45]. The stress varies strongly along the entire scan track.

**Figure 9:**
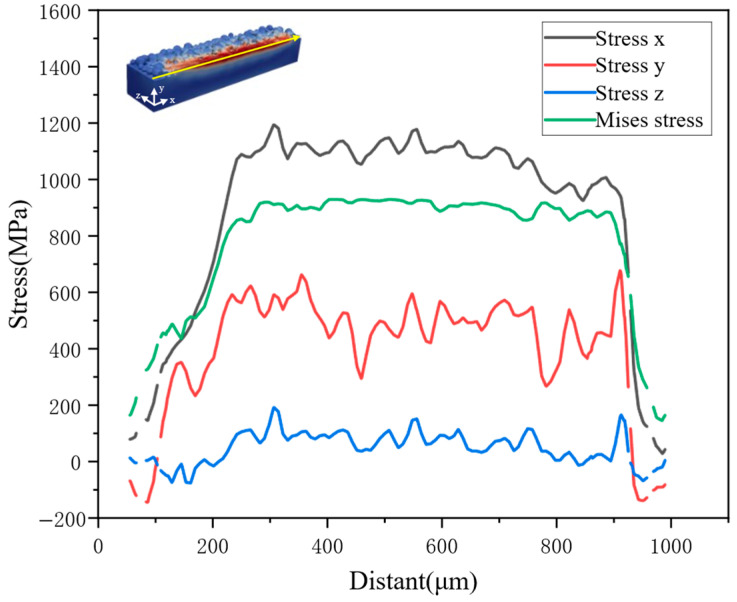
The stress along the middle of the melt track.

A more detailed investigation is performed along the centerline of the melt track, and the results are shown in Figure 9. The stress in the x-direction is higher than in the other two directions, and the maximum value reaches 1200 MPa. The stresses in the three directions are consistent with the von Mises equivalent stress, and all exhibit periodic oscillations with a wavelength of approximately 200 μm. Such periodic behavior may be related to cyclic laser heating and natural cooling, which cause local temperature gradients, solidification positions, and free surfaces of the melt pool to oscillate periodically and consequently give rise to periodic variations in solidification shrinkage and residual stress [46,47]. Comparison with the track surface shows that stress evolution is strongly correlated with the weld-bead morphology: stress peaks occur at the low-lying regions of the bead, which may be due to thinner deposited layers and higher cooling rates in these regions.

**Figure 10:**
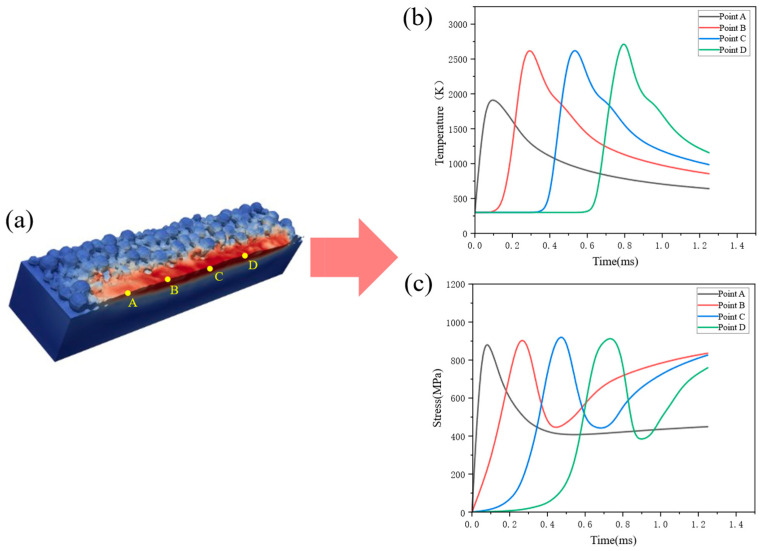
Four observation points along the melt track: (**a**) positions; (**b**) temperature histories; (**c**) stress histories.

To investigate stress evolution along the scan path, four points (A, B, C, and D) are selected along the laser track from the start position, as shown in Figure 10a, and the temporal evolution of their temperature and stress is recorded. The four points exhibit similar temperature and stress evolution patterns. The peak temperature at point A is lower than those at the other three points, reaching about 1900 K, whereas points B, C, and D have peak temperatures of approximately 2750 K. This is because point A is located near the beginning of the scan, where the residence time of the heat source is relatively short. After scanning, the cooling rates from the peak temperature increase from 300 K/ms to 20,000 K/ms for points A, B, C, and D, respectively. Correspondingly, the rate of increase in residual stress during cooling rises from 58.8 MPa/ms to 1000 MPa/ms. In addition, slight temperature fluctuations are observed during cooling, which are likely related to melt-pool flow. It should be noted that the present study uses a small numerical time step so that the temperature increment in each iteration is strictly controlled, enabling the resolution of steep temperature gradients and solidification processes associated with very rapid cooling while avoiding numerical oscillations. However, the current model does not incorporate microstructural kinetics of non-equilibrium solidification; hence the influence of rapid cooling on stress evolution is captured only through the thermo-elastoplastic framework.

It is also observed that the stress reaches a local maximum of about 900 MPa before the temperature peaks and then drops abruptly to approximately 400 MPa. This may be because, when the laser focus is located near the observation point, powder particles are in a semi-molten state and the laser power is high, leading to a lower pressure at the center of the heat source. A pressure difference is formed between the observation point and the center, causing powder to be drawn toward the heat source and subject to large stresses. Subsequently, the arrival of the heat source melts the point, reducing the stress. As the heat source moves away, the temperature decreases, the melt pool solidifies and shrinks, and the stress increases again.

**Figure 11:**
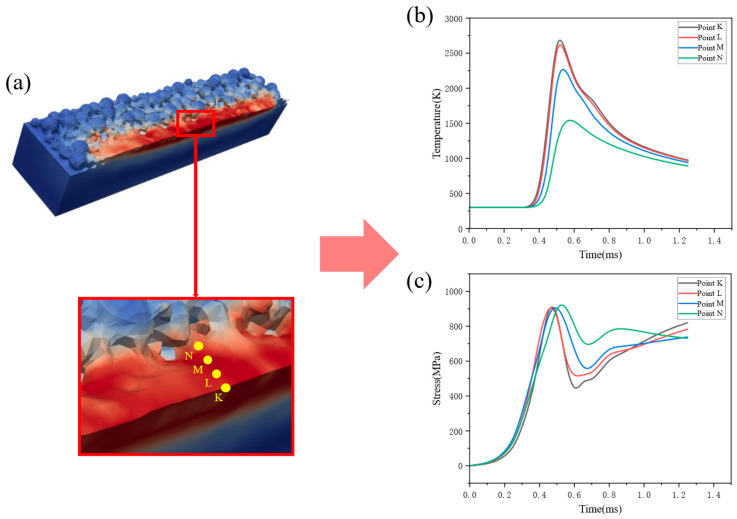
Our observation points across the melt-pool width: (**a**) positions; (**b**) temperature histories; (**c**) stress histories.

Furthermore, four observation points K, L, M, and N are selected along a line perpendicular to the weld bead, as shown in Figure 11a. The temperature and stress histories at these points are shown in Figure 11b,c. Points K and L lie on the melt track and reach a maximum temperature of 2750 K; point M is located at the fusion line with a peak temperature of 2250 K, and point N lies in the heat-affected zone with a peak temperature of only 1350 K. The cooling rates of points K, L, M, and N are approximately 2692 K/ms, 2461 K/ms, 1923 K/ms, and 769 K/ms, respectively. Because points K and L lie within the melt track, they cool faster and have higher residual stresses of about 800 MPa, whereas points M and N cool more slowly and have lower residual stresses of about 700 MPa.

### 4.2. Comparison with the Conventional Birth–Death Method

**Figure 12:**
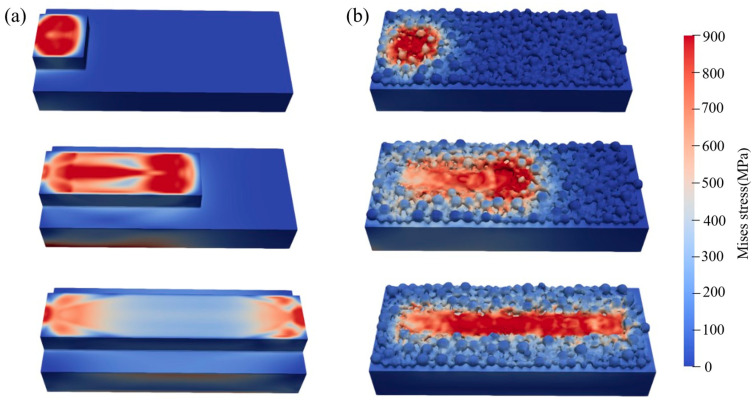
Comparison of simulation results: (**a**) conventional birth–death method; (**b**) proposed algorithm.

To highlight the differences between the proposed algorithm and the conventional birth–death method, SLM simulations are also performed using the traditional birth–death approach with the same model dimensions and laser parameters. The simulation results are compared in Figure 12. It can be seen that, compared with the traditional method, the proposed algorithm significantly improves the accuracy of the simulated powder-bed morphology and more realistically reproduces the melt-pool geometry. In the traditional model, the stress distribution is essentially symmetric about the centerline of the melt pool, whereas the proposed model, by incorporating melt-pool flow and free-surface evolution, captures a more complex and realistic stress distribution in SLM.

**Figure 13:**
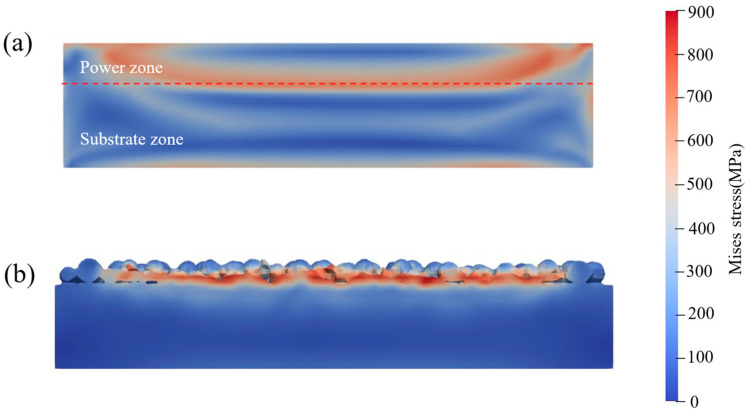
Comparison of simulation result cross-sections: (**a**) Traditional birth and death element algorithm; (**b**) Proposed algorithm.

To examine the internal stress distribution, cross-sections parallel to the melt track at the same position are extracted from the results of both methods, as shown in Figure 13. In the traditional model, the stress distribution is approximately axisymmetric, whereas in the proposed model, the stress distribution varies with the surface morphology and better reflects the complex reality in SLM. Furthermore, the proposed model reveals pores and lack-of-fusion regions that cannot be observed in the traditional model; these defects are crucial in real SLM processes. In the traditional model, the region of high stress forms a wide “V”-shaped band with a maximum stress of about 650 MPa and relatively low stress on the surface. In contrast, in the proposed model, the high-stress region is mainly concentrated near the surface and shallow subsurface of the melt pool, with a maximum stress of about 900 MPa, which may be attributed to melt-pool flow effects.

The total computation time of the traditional birth–death method is approximately 6 h, whereas that of the coupled algorithm is about 12 h, i.e., the computational cost is roughly doubled. Although the coupling scheme is more expensive than the conventional method, it significantly improves the physical consistency of stress and surface-morphology prediction. The traditional birth–death model typically assumes a fixed melt-pool geometry and a simplified deposition process without considering the effects of melt-pool flow and free-surface evolution on stress and is therefore more suitable as a fast engineering approximation. In contrast, the coupled algorithm naturally captures the evolution of temperature gradients and free-surface morphology during rapid solidification, thereby providing a more accurate description of stress concentration, defect formation, and their spatial distribution.

### 4.3. Influence of Surface Quality on Residual Stress

**Figure 14:**
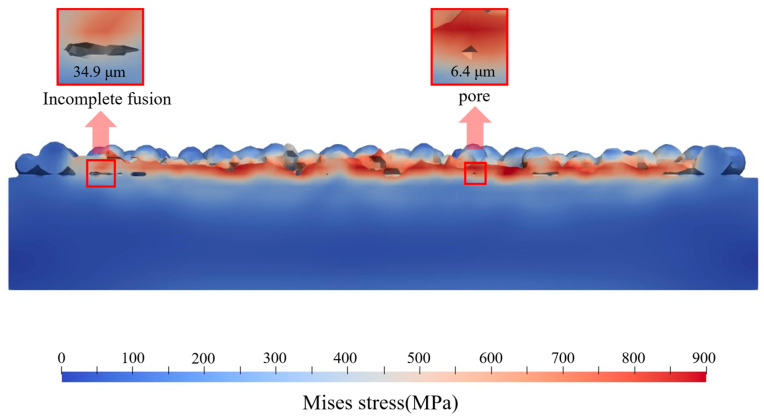
Stress distribution around surface defects.

Owing to its high fidelity, the present model can resolve lack-of-fusion and pore defects. As shown in Figure 14, the size of the lack-of-fusion defect reaches 34.9 μm and that of the pore defect reaches 6.4 μm. Although these defects are relatively small, their distributions and orders of magnitude are consistent with typical defect sizes reported in the literature [31,48,49]. Observation of cross-sections cut across the melt track indicates that high residual stresses occur around these defects: the stress concentration near the lack-of-fusion region reaches about 700 MPa, whereas that around the pore reaches about 900 MPa.

**Figure 15:**
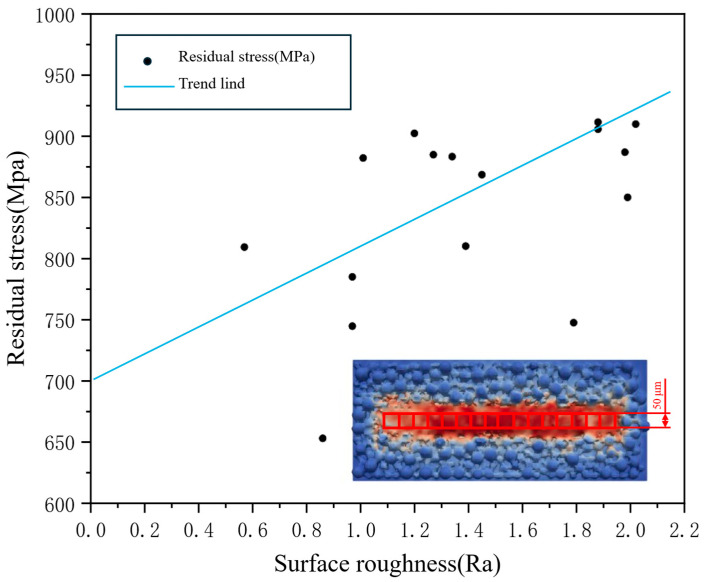
Relationship between surface roughness and residual stress.

To investigate the relationship between local free-surface morphology and residual stress, a uniform region of 50 μm × 50 μm is selected within the melt track, and the surface roughness and residual stress in this region are statistically analyzed, as shown in Figure 15. The results show that locations with more pronounced surface undulations correspond to higher residual stresses, with the maximum residual stress reaching approximately 910 MPa, whereas regions with relatively smooth morphology exhibit lower residual stresses, with a minimum of about 650 MPa. This indicates a positive correlation between surface roughness and local stress concentration. Previous studies have reported that melt-pool flow instabilities and non-uniform solidification paths in SLM hinder uniform release of thermal strains, leading to higher local residual stresses in areas with larger surface waviness [47,49]. These findings are consistent with the conclusions of the present work. It should be emphasized that the conclusions drawn here aim to elucidate the qualitative relationship between surface roughness and residual stress. The trend line in Figure 15 is only used to indicate the observed trend in this study and is not intended as a universal quantitative law.

## 5. Conclusions

(1) Based on our previous work, a CFD-FEM multiphysics coupling framework is introduced for predicting stress distribution in micrometer-scale SLM. The CFD model is used to solve melt-pool flow, transient temperature, and free-surface evolution, and a dynamic interface-element method is employed to achieve continuous transfer of morphology and temperature data within the FEM model. By adjusting the mesh size so that the nodes in the CFD and FEM models correspond one-to-one, the accuracy of data mapping is improved, and the prediction of SLM residual stresses is enhanced.
(2) Compared with the conventional birth–death method, the proposed coupling framework provides much higher fidelity in predicting defects and surface morphology. Micrometer-scale single-track SLM simulations performed using the coupled model show that local residual stresses can reach about 900 MPa and that the sizes of pore and lack-of-fusion defects are approximately 6.4 μm and 34.9 μm, respectively. A clear positive correlation between surface roughness and stress concentration is observed.
(3) Although the computational cost of the coupled algorithm is about twice that of the traditional birth–death method, it yields significantly more accurate predictions of the spatial distribution of residual stress, free-surface morphology, and defect sizes. Therefore, it is well-suited for mechanistic studies of stress concentration and defects in SLM, as well as for component design and process optimization. In contrast, the traditional birth–death method remains suitable for rapid engineering estimates.

## Figures and Tables

**Table 1:** Laser parameters.

Laser Power (W)	Scanning Speed (mm/s)	Spot Radius (mm)
150	800	0.07

**Table 2:** TC4 alloy parameters.

Parameters	Values
Molar mass	0.04748 kg/mol
Latent heat of evaporation	9830 kJ/kg
Density	4430 kg/m^3^
Liquidus temperature	1878 K
Solidus temperature	1928 K
Evaporating temperature	3315 K
Liquid metal viscosity	0.0019 kg/m/s
Coefficient of surface tension	1.63 N/m
Latent heat of fusion	440 kJ/kg
Thermal capillarity coefficient	−0.00026 N/m/K

## Data Availability

The original contributions presented in this study are included in the article. Further inquiries can be directed to the corresponding author.
